# Stable Isotope Dilution Assays for Clinical Analyses of Folates and Other One-Carbon Metabolites: Application to Folate-Deficiency Studies

**DOI:** 10.1371/journal.pone.0156610

**Published:** 2016-06-08

**Authors:** Markus Kopp, Rosalie Morisset, Peter Koehler, Michael Rychlik

**Affiliations:** 1 Chair of Analytical Food Chemistry, Technische Universität München, Alte Akademie 10, D-85354 Freising, Germany; 2 Institute for Food & Health (Z I E L), Technische Universiät München, Weihenstephaner Berg 1, D-85354 Freising, Germany; 3 Chair of Nutritional Physiology, Technische Universität München, Gregor-Mendel-Straße 2, D-85354 Freising, Germany; 4 Deutsche Forschungsanstalt für Lebensmittelchemie, Leibniz Institut, Lise-Meitner-Straße 34, D-85354 Freising, Germany; Michigan State University, UNITED STATES

## Abstract

Folate deficiency is generally accepted as a potential direct or indirect risk factor for diseases including spina bifida, coronary heart diseases, malfunctions of the central nervous system, and cancer. The direct inclusion of folates in the methylation cycle, including the remethylation of homocysteine and regeneration of S-adenosylmethionine, underlines the importance of these vitamins and other components of one-carbon metabolism. Therefore, the aim of the present study was to develop a multiple stable isotope dilution assay (SIDA) for the respective analytes in plasma and tissue samples to allow for a closer look at the interaction between a severe folate deficiency and local folate status, as well as further interactions with circulating S-adenosylmethionine, S-adenosylhomocysteine, and homocysteine. The analytical methods were based on SIDAs coupled with liquid chromatography—tandem mass spectrometry (LC-MS/MS) analysis using the deuterated folates [^2^H_4_]-5-methyltetrahydrofolic acid, [^2^H_4_]-5-formyltetrahydrofolic acid, [^2^H_4_]-tetrahydrofolic acid, [^2^H_4_]-10-formylfolic acid, and [^2^H_4_]-folic acid and the deuterated one-carbon metabolites [^2^H_4_]-homocysteine, [^2^H_4_]-S-adenosylhomocysteine, and [^2^H_3_]-S-adenosylmethionine as internal standards. Three analytical methods have been developed for the analysis of homocysteine, S-adenosylmethionine, S-adenosylhomocysteine, and six folate vitamers. Validation data for the analysis of C_1_-metabolites in plasma and tissue samples or folate analysis in tissue samples revealed excellent sensitivity, precision, and recovery for all analytes studied. The miniaturized methods using sample volumes as low as 50 μL and weighed portions of 5–25 mg will allow the assessment of the status of folates and additional biomarkers of impaired one-carbon metabolism during folate deficiency.

## Introduction

Folate deficiency and alterations in one-carbon metabolism are considered to be potential risk factors for several diseases such as neural tube defects in newborns [1,2], cardiovascular diseases [3], Alzheimer’s disease [4,5], and certain forms of cancer [6,7]. Recent scientific reports describe the essentiality of an appropriate daily intake of folate vitamers for the preservation of health [2,8]. For assessing the folate status and the methylation capacity during folate deficiency in a mouse model, several marker compounds have to be evaluated. Plasma folate analysis is typically used to determine short-term supply and folate bioavailability, e.g., in food [9], thereby representing the state of absorption after uptake, utilization, and storage [10]. These fluctuations cause it to be an unsuitable biomarker for folate status [10] and repeated long-term analysis of fasting plasma levels has to be carried out for its determination [8]. Erythrocyte folate mirrors the supply present during a period of approximately four months preceding the measurement [11] as red blood cells retain folates as a possible result of hemoglobin binding [12]. Therefore, it reflects the folate intake over a certain time period and is accepted as a suitable marker for intermediate folate status. For the analysis of erythrocyte folate, methods using <50 mg of erythrocytes have been published [13]. Whereas the kidney seems to play a crucial role in vitamin homeostasis [14], the liver is considered as the main folate storing tissue [15]. Of the presumed 10–100 mg whole body folate, 3–16 mg are stored in the liver [16,17]. As the brain and heart are involved in the outcome of some of the aforementioned diseases, these additional organs may be included in methods for the assessment of folate status and distribution. Homocysteine (Hcy), particularly its role as a marker or causal agent for diseases, is still discussed controversially. The interrelation between Hcy and the development of arteriosclerosis has not yet been clarified and some “complex pathways of inflammation have been suggested” [18]. Recent studies revealed a potential correlation between Hcy and the development of small-vessel disease in the brain [18,19]. Moreover, Hcy has been reported as a marker for the diagnosis of vitamin B_12_ and folate deficiency as both vitamins are directly involved in the methylation of homocysteine to methionine, which is mediated by the methionine synthase reaction. Besides vitamin B_12_ or folate deficiency, inborn metabolic defects leading to homocystinuria or hyperhomocysteinemia also have to be taken into account. These defects include either the trans-sulfuration or transmethylation pathways [20]. The most common form of homocystinuria is attributed to an inborn error leading to cystathionine-β-synthase deficiency [20,21]. Nevertheless, Hcy is also influenced by age, sex, lifestyle, and further genetic determinants [21]. Therefore, an elevated Hcy level in plasma is a meaningful diagnostic parameter for B_12_ or folate status only in combination with the determination of further relevant markers for the latter. To characterize the chronological development of metabolic changes during depletion of folate stores and decreased methylation capacity in selected tissues, the tissue levels of S-adenosylmethionine (AdoMet) and S-adenosylhomocysteine (AdoHcy), which are directly involved in the methylation circle, have to be included in addition to Hcy concentration in plasma and folate status in erythrocytes. AdoMet is a ubiquitous donor of methyl-groups and is directly involved in the methylation of DNA, proteins, phospholipids, and neurotransmitters [22]. The AdoMet/AdoHcy ratio reflects the tissue-specific cellular methylation capacity [23,24], whereas plasma AdoMet and AdoHcy are not considered as appropriate markers for methylation capacity in tissue because there is no equal exchange of both compounds with other biological units [24].

Although microbiological assays are the conventional tools for folate analysis, substantial progress has been achieved in the analysis of Hcy, AdoMet, and AdoHcy in plasma by establishing stable isotope dilution assays (SIDAs) conducted as LC-MS/MS measurements and decreasing the sample amount applied for extraction [25–28]. However, data availability on the distribution of AdoMet or AdoHcy in tissue is poor [29–33].

In folate analysis, SIDAs have proven their advantage over standard methodologies. Thus, our primary focus was to design a suitable multi SIDA for the quantification of six folate vitamers, including four bioactive forms (5-formyltetrahydrofolic acid (5-CHO-H_4_folate), tetrahydrofolic acid (H_4_folate), 5-methyltetrahydrofolic acid (5-CH_3_-H_4_folate), and 5,10-methenyltetrahydrofolic acid (5,10-CH^+^-H_4_folate)), two oxidized forms represented by 10-CHO-folate and folic acid, and two additional one-carbon metabolites including AdoMet and AdoHcy in one tissue sample and tHcy in plasma. Moreover, we focused on the reduction of sample amounts to enable further analyses of the remaining tissue matter.

As folates are unstable when exposed to UV-light [34] and oxygen [35], and AdoMet and AdoHcy concentrations are easily affected by enzymatic interconversion [36], two different extraction procedures are needed to meet the requirements of both analyte groups in tissue. Folate analysis requires an enzymatic deconjugation step to determine folate patterns in their monoglutamylated form [13], whereas enzymatic activity during the extraction of AdoHcy and AdoMet has to be prevented immediately [36]. Furthermore, a suitable tissue sampling technique has to be designed to ensure the stability of all analytes after repeated freeze-thaw cycles without enzymatic interconversion or degradation. For the determination of plasma Hcy (quantified as total homocysteine, tHcy) a reduction step with dithiothreitol (DTT) is required [37] to reduce the disulfide bonds of oxidized Hcy occurring mainly as protein-bound Hcy (~80%) or cysteine-Hcy dimers and homocystine (~20%) [38]. Only ~1% of tHcy can be attributed to the free thiol [38].

## Materials and Methods

### Chemicals

Rat serum and chicken pancreas containing γ-glutamyl hydrolase (EC 3.4.19.9) were obtained from Biozol (Eching, Germany) and Difco (Sparks, MD, USA), respectively. Acetonitrile, potassium dihydrogen phosphate, disodium hydrogen phosphate (anhydrous), methanol, sodium chloride, sodium acetate trihydrate, and sodium hydroxide were purchased from Merck (Darmstadt, Germany). Dithiothreitol (DTT) was purchased from Applichem Lifescience (Darmstadt, Germany). Isoflurane was obtained from Baxter (Deerfield, USA). Formic acid, 4-morpholineethanesulfonic acid hydrate (MES), AdoMet p-toluenesulfonate, AdoHcy, Hcy, and Triton X-100 were obtained from Sigma (Deisenhofen, Germany). Ascorbic acid was obtained from VWR Chemicals Prolabo (Leuven, Belgium). The isotopological standards [^2^H_4_]-5-CH_3_-H_4_folate, [^2^H_4_]-5-CHO-H_4_folate, [^2^H_4_]-10-CHO-folate, [^2^H_4_]-H_4_folate, and [^2^H_4_]-folic acid were synthesized as reported recently [39]. [^2^H_4_]-Hcy and [^2^H_3_]-AdoMet were purchased from C/D/N Isotopes (Pointe-Claire, Quebec, Canada). [^2^H_4_]-AdoHcy was obtained from Cayman Chemicals (Ann Arbor, MI, USA). 5-CH_3_-H_4_folate, 10-CHO-folate, 5-CHO-H_4_folate calcium salt, H_4_folate trihydrochloride, and 5,10-CH^+^-H_4_folate chloride were purchased from Schircks Laboratories (Jona, Switzerland). Folic acid was obtained from Fluka (Buchs, Switzerland). Strata SAX^®^ cartridges (quaternary amine, 100 mg, 1 mL) were obtained from Phenomenex (Aschaffenburg, Germany). Microvette^®^ 500 K3E tubes were purchased from Sarstedt (Nümbrecht, Germany). NIST 1955 Standard Reference Material was purchased from LGC Standards (Wesel, Germany). For the lyophilization step a Christ alpha 1–2 LD plus freeze dryer (Osterode, Germany) was used. Homogenization of tissue samples was carried out with a TissueRuptor^®^ homogenizer from Qiagen (Hilden, Germany).

### Solutions

For tissue folate determination, the following buffers and solutions were prepared. Extraction buffer 1 consisted of a 20 g/L solution of ascorbic acid and 200 mmol/L MES with 6.5 mmol/L DTT adjusted to pH 5 with 7.5 M sodium hydroxide. Extraction buffer 2 comprised 0.1% Triton X-100 dissolved in extraction buffer 1. Phosphate buffer (100 mmol/L) was prepared by adjusting an aqueous solution of 100 mmol/L disodium hydrogen phosphate with an aqueous solution of potassium dihydrogen phosphate (100 mmol/L) to pH 7.0. The equilibration buffer (10 mmol/L) for solid phase extraction (SPE) was prepared by tenfold dilution of phosphate buffer with 1.3 mmol/L DTT as additive. Elution was performed with a mixture of sodium chloride (5%) and 100 mmol/L aqueous sodium acetate containing 6.5 mmol/L DTT and ascorbic acid (1%). Chicken pancreas suspension for folylpolyglutamate deconjugation was prepared by stirring chicken pancreas (30 mg) in aqueous phosphate buffer solution (90 mL, 100 mmol/L) containing 1% ascorbic acid adjusted to pH 7 with 7.5 mol/L sodium hydroxide. The suspension was stored at −20°C until use. Rat serum was thawed prior to extraction. For tissue AdoMet and AdoHcy determination, the following solutions were prepared. Dilution solvent 1 for AdoMet and AdoHcy consisted of 0.1% (v/v) formic acid in water/acetonitrile (98/2 v/v). Dilution solvent 2 consisted of 6.5 mmol/L DTT in water/acetonitrile (98/2 v/v) with 0.1% (v/v) formic acid. The precipitation reagent consisted of 100 mmol/L DTT in methanol/water (90/10 v/v) with 0.1% (v/v) formic acid. Reagents for tHcy extraction consisted of 200 mmol/L aqueous DTT (incubation reagent) and methanol as precipitation reagent.

### Sampling procedure

For the preclinical study, C57BL/6N mice (female, 9 weeks of age) were used. All animal procedures were approved by the Bavarian Animal Care and Use Committee and all experiments were performed in accordance with relevant guidelines and regulations. Mice were bred and kept in animal facilities of the Institute for Food and Health (ZIEL). All animals had free access to water and were fed standard chow (V1534 R/M-H; ssniff Spezialdiäten GmbH, Germany). They were not fasted prior to sampling of blood and tissues. For blood and tissue sampling, mice were sacrificed by isoflurane overdose. Blood was collected into EDTA-coated tubes via cardiac puncture and centrifuged for 10 min at 1200 g and 4°C to separate plasma. Plasma and tissues were collected, thereafterflash-frozen instantaneously in liquid nitrogen, and stored at -80°C until use. All tissue samples, including liver, heart, kidney, and brain, were lyophilized for 24 h and subsequently homogenized with a micro pestle at room temperature to avoid condensation in the dried tissue powder. For validation, tissue samples were pooled for each organ. Aliquots of 10 mg of lyophilized liver, kidney, and heart and 20 mg of lyophilized brain were prepared for AdoMet and AdoHcy analyses in centrifuge tubes. Furthermore, 5 mg of freeze-dried liver, 10 mg of freeze-dried kidney, and 25 mg of lyophilized brain and heart were prepared in centrifuge tubes for folate extraction. All extracts were stored at −20°C until LC-MS/MS measurement.

### Folate in liver, brain, heart, and kidney

9.5 ng of [^2^H_4_]-5-CH_3_-H_4_folate, 6.1 ng of [^2^H_4_]-H_4_folate, 7.5 ng of [^2^H_4_]-5-CHO-H_4_folate, 1.7 ng of [^2^H_4_]-10-CHO-folate, and 2.1 ng of [^2^H_4_]-folic acid were added to the tissue samples. After adding 2 mL of extraction buffer 2, samples were homogenized for 10 s with a TissueRuptor^®^ and placed in a chilled ultrasonic bath for lysis for 30 min. Aliquots of 150 μL of rat serum and 1 mL of chicken pancreas suspension were added, and the suspension was incubated for 4 h at 37°C under constant agitation in a water bath. After heating for 4 min at 100°C, samples were cooled in an ice bath and centrifuged at 2700 g and 4°C for 20 min.

### Solid-phase extraction (SPE)

All extracts were purified by SPE using a 12-port manifold (Merck, Darmstadt, Germany) equipped with Strata SAX cartridges (quaternary amine, 100 mg, 1 mL). The stationary phase was activated with two column volumes of methanol and two column volumes of equilibration buffer followed by the application of the respective tissue extracts. Afterwards, the cartridges were washed with two column volumes of equilibration buffer and subsequently dried by evaporation in vacuo. Elution was carried out with 0.5 mL eluting solution.

### AdoMet and AdoHcy in liver, brain, heart, and kidney

Aliquoted tissue samples were placed in a freezing mixture (-80°C) consisting of dry ice in acetone. Protein precipitation was carried out with 1.5 mL precipitation reagent, and 611 ng of [^2^H_3_]-AdoMet and 77.5 ng of [^2^H_4_]-AdoHcy were added to the lyophilized samples. The suspension was then homogenized for 30 s with a TissueRuptor^®^, subsequently incubated for 15 min at 4°C, and placed in the freezing mixture again. After centrifugation at 2700 g and 4°C for 20 min, the supernatant was removed and dried under a nitrogen flow. The pellet was resuspended in 500 μL of dilution solvent 1, vortex-mixed, and centrifuged for 3 min at 15400 g. Aliquots of 200 μL of the supernatant were transferred into an autosampler vial for LC-MS/MS analysis.

### tHcy in plasma

Aliquots of 50 μL of mouse plasma were spiked with 30 ng of [^2^H_4_]-Hcy and vortex-mixed. After 15 min of equilibration, 50 μL of a 200 mmol/L aqueous DTT solution was pipetted into the plasma. Following further vortex-mixing, the sample was incubated for 30 min at ambient temperature and subsequently mixed with 0.35 mL of methanol for protein precipitation. After an additional 10 min in the freezer, the plasma was vortex-mixed, and the supernatant was removed by centrifugation at 15400 g for 3 min and subsequently dried under nitrogen flow. The pellet was resuspended in 250 μl of dilution solvent 1, vortex-mixed, and centrifuged at 15400 g for an additional 3 min. Two hundred microliters were transferred into an autosampler vial for LC-MS/MS analysis.

### Optimization and validation

#### Influence of the homogenization procedure on AdoMet, AdoHcy and H_4_folate levels

A flash frozen liver sample was divided into three similar pieces (left, medial, right) and homogenized as described above after lyophilization (medial and right pieces) or by using a precooled mortar and pestle immersed in liquid nitrogen (left piece). All samples were prepared in duplicate. After extraction of 10 mg of lyophilized and 100 mg of fresh liver, AdoMet and AdoHcy were evaluated using LC-MS/MS. An additional 5 mg of lyophilized and 100 mg of fresh liver were extracted and analyzed for their H_4_folate content.

#### Optimization of folate extraction

Three aliquots in triplicate (i.e., nine aliquots in total) of 5 mg lyophilized mouse liver were spiked with 9.5 ng of [^2^H_4_]-5-CH_3_-H_4_folate, 6.1 ng of [^2^H_4_]-H_4_folate, and 7.5 ng of [^2^H_4_]-5-CHO-H_4_folate and extracted as detailed above to evaluate folate interconversion during extraction. A first aliquot (i) was extracted in triplicate with 2 mL of extraction buffer 1 (without Triton X-100) and equilibrated for 15 min after homogenization with a TissueRuptor^®^ followed by enzymatic treatment and extraction as aforementioned. A further aliquot was suspended in triplicate and homogenized with a TissueRuptor^®^ in extraction buffer 2 (containing Triton X-100) and placed in a chilled ultrasonic bath for cell lysis for 30 min (ii). The remaining tissue samples (third aliquot in triplicate) were homogenized in extraction buffer 1, heated for 5 min in a water bath, and chilled on ice prior to enzymatic treatment (iii).

#### Influence of DTT and formic acid on AdoMet during extraction and detection

Six aliquots of 10 mg liver surrogates were each spiked with 0.7 μg AdoMet. Three different mixtures were used as precipitation agents. Besides the precipitation reagent mentioned above for standard extraction, methanol/water (90/10 v/v) (i) was prepared with 0.1% (v/v) formic acid (ii) and without formic acid and DTT (iii). Two aliquots of spiked matrix were each suspended in solvent (i), (ii), and (iii) for protein precipitation and extracted according to the standard protocol for tissue samples. The deuterated internal standard was added after extraction. Recoveries of AdoMet in the spiked surrogates were calculated by comparing the concentrations of (i)–(iii) obtained with the initial concentration of the stock solution (100%).

#### Optimal DTT concentration for tHcy analysis

To evaluate the concentration of DTT necessary for total reduction of bound Hcy, two solutions of DTT were prepared in water. Two aliquots of 50 μL of rat serum were incubated with 50 μL of either 200 or 400 mmol/L aqueous DTT as described above and analyzed for tHcy.

#### Applicability of the multi stable isotope dilution assay

To confirm the applicability of all methods, three female C57BL/6N mice (9 weeks) were examined for their folate, AdoMet, and AdoHcy status in brain, heart, kidney, and liver, and tHcy in plasma.

#### LC-MS/MS

Folates in tissue extracts were determined by means of LC-MS/MS (Finnigan Surveyor Plus HPLC System, Thermo Electron Corporation, Waltham, USA; triple quadrupole TSQ quantum discovery mass spectrometer, Thermo Electron Corporation, Waltham, USA). The vitamers were separated on a YMC Pack Pro C_18_ column (150 × 3 mm, 3 μm, YMC, Kyoto, Japan). The mobile phase for gradient elution consisted of 0.1% (v/v) aqueous formic acid (eluent A) and acetonitrile containing 0.1% (v/v) formic acid (eluent B) at a flow rate of 0.3 mL/min. Gradient elution started at 5% B, followed by a linear increase of B to 10% within 5 min. After holding for 5 min, B was increased to 15% within 10 min and to 50% within 2 min and held for 2 min. Subsequently, the mobile phase was programmed to 90% B within two min. After 4 min, B was decreased to 5% within 2 min before equilibrating the column for 14 min with the initial solvent mixture. Ion scanning was performed from 5 to 23.5 min. The spectrometer was operated in the positive electrospray ionization mode using selective reaction monitoring (SRM). The spray voltage was set to 3900 V, capillary temperature to 320°C, and the capillary voltage to 35 V. The oven temperature was set to 30°C. The SRM scan parameters are shown in Table 1.

**Table 1:** SRM scan parameters for the folate vitamers and the respective internal standard (TSQ).

Analyte	Q1 (m/z)	Q3 (m/z)	CE	CID
5-CH_3_-H_4_folate	460	313	21	10
5,10-CH^+^-H_4_folate	456	412	30	8
H_4_folate	446	299	19	10
Folic acid	442	295	19	10
10-CHO-folate	470	295	19	10
5-CHO-H_4_folate	474	327	25	10
[^2^H_4_]-5-CH_3_-H_4_folate	464	317	21	10
[^2^H_4_]-H_4_folate	450	303	19	10
[^2^H_4_]-Folic acid	446	299	19	10
[^2^H_4_]-10-CHO-folate	474	299	19	10
[^2^H_4_]-5-CHO-H_4_folate	478	331	25	10

m/z: mass/charge ratio, CE: Collision energy, CID: Collision induced dissociation.

The system for Hcy, AdoMet, and AdoHcy measurement consisted of a Shimadzu Prominence LC-20A System (Shimadzu, Kyoto, Japan) and an API 4000 Q-Trap mass spectrometer (AB Sciex, Foster City, CA, USA). Analyte separation was carried out on a Phenomenex Gemini reversed phase column (110A 3u, 150 × 4.60 mm, Phenomenex, Aschaffenburg, Germany). The mobile phase for gradient elution consisted of 0.1% (v/v) aqueous formic acid (eluent A) and acetonitrile containing 0.1% (v/v) formic acid (eluent B) at a flow of 0.4 mL/min. Starting conditions were 2% of solvent B held for 3 min. Following a linear increase to 100% B within 6.5 min, B was set to the starting conditions within 5 min after a 3 min hold at 100%. Column equilibration was carried out for 11.5 min resulting in a total run time of 29 min. Ion scanning was performed from 2 to 12 min. The spectrometer was operated in the positive electrospray mode using multiple reaction monitoring (MRM). The ion source was set to 5000 V, the interface heater to 500°C, and the oven temperature was set to 30°C. The MRM scan parameters are shown in Table 2.

**Table 2:** MRM scan parameters for AdoHcy, AdoMet, Hcy, and the respective internal standards (API 4000).

Compound	Q1 (m/z)	Q3 (m/z)	DP	CE	CXP
Hcy	136.2	90.0	36	15	6
	136.2	118.0	36	11	6
[^2^H_4_]-Hcy	140.2	94.0	36	15	6
	140.2	122.0	36	11	6
AdoHcy	385.2	136.1	51	27	6
	385.2	250.0	51	19	12
[^2^H_4_]-AdoHcy	389.2	138.1	51	27	6
	389.2	254.2	51	19	12
AdoMet	399.2	250.0	51	21	12
	399.2	136.1	51	33	6
[^2^H_3_]-AdoMet	402.2	250.0	51	21	12
	402.2	136.1	51	33	6

DP: Declustering potential, CE: collision energy, CXP: Cell exit potential.

#### Quantitative NMR (qNMR)

Purities of one-carbon metabolites were determined by qNMR experiments prior to calibration with a Bruker Avance III UltraShield 400 MHz NMR Spectrometer (Bruker, Billerica, Mass., United States).

#### LC-DAD

The purities of unlabeled folate standards used for calibration of SIDAs were determined by measuring the analyte with the HPLC-DAD method previously published with minor modifications [40]. The wavelength of detection was λ_max_ = 290 nm for 5-CH_3_-H_4_folate and 5-CHO-H_4_folate and λ_max_ = 272 nm for H_4_folate, 5,10-CH^+^-H_4_folate, and 10-CHO-folate. Calibration standards were prepared by the dilution of folate vitamers in extraction buffer 1 after dissolving in phosphate buffer. The folic acid (23 nmol) internal standard (46 nmol for 5,10-CH^+^-H_4_folate) was mixed with twentyfold to onefold amounts of analytes for calibration. The analyte stock solution was used for further dilution and calibration.

Analyte separation for the one-carbon metabolites was carried out on a Phenomenex Gemini reversed phase column (110A 3u, 150 × 4,60 mm, Phenomenex, Aschaffenburg, Germany). The wavelength of detection was λ_max_ = 260 nm for AdoMet, AdoHcy, and adenosine (internal standard). The mobile phase consisted of 0.1% (v/v) aqueous formic acid (eluent A) and 0.1% (v/v) formic acid in acetonitrile (eluent B). The gradient started with 2% B for 2 min and increased linearly to 100% B within 11 min. After 5 min, B was decreased to 2% over 4 min, and the column was equilibrated for 5 min. Aliquots of adenosine (78 nmol) were mixed with the analyte in molar ratios between 2:1 and 1:5 and dissolved in dilution solvent 1 for calibration. Analyte stock solutions of AdoMet and AdoHcy were used for further dilution and calibration. Hcy calibration solutions were prepared gravimetrically by dissolving Hcy in water/acetonitrile (98/2; v/v) and further dilution with dilution solvent 2.

#### Calibration and quantitation

Folate calibration solutions were prepared by mixing the internal standard solution with the corresponding analyte solutions. Molar ratios of internal standard (S) and analyte (A) [n(S)/n(A)] for the calibration were 7:1–1:10, 3:1–1:76, 8:1–1:100, 16:1–1:32, 1:1–1:500, 36:1–1:10, 14:1–1:24, 11:1–1:15, and 3:1–1:30 for folic acid, 5-CH_3_-H_4_folate, 10-CHO-folate, 5-CHO-H_4_folate, H_4_folate, 5,10-CH^+^-H_4_folate, AdoMet, AdoHcy, and Hcy, respectively. 5,10-CH^+^-H_4_folate was calculated with [^2^H_4_]-5-CH_3_-H_4_folate as the internal standard. For the calibration functions, linear regression was used by combining the molar ratios with the peak area ratios [A(S)/A(A)] obtained from LC-MS/MS analysis. Linearity of the calibration functions was confirmed by the Mandel-Test for all analytes except for 5,10-CH^+^-H_4_folate. However, for the latter, a linear function was also used as the quantitation of 5,10-CH^+^-H_4_folate has to be considered less accurate than that for the other analytes because of its fast interconversion to 5-CHO-H_4_folate and the use of non-analogous standard [^2^H_4_]-5-CH_3_-H_4_folate. However, this was still acceptable as the interconverted 5,10-CH^+^-H_4_folate was quantified as 5-CHO-H_4_folate. Consistency of response was verified by injecting a randomly chosen n(S)/n(A) mixture in the linear range of the response functions.

#### Limits of detection (LODs) and quantification (LOQs)

LODs and LOQs for folates and one-carbon metabolites were determined in modified pig tissue from a local butcher or in matrix surrogate. AdoMet was determined in processed liver, kidney, and brain. All tissue samples were homogenized in a standard mixer and stirred in 5% hydrogen peroxide in a water bath at 60°C. Hydrogen peroxide was quenched by adding aqueous ascorbic acid followed by repeated testing for peroxide residues. Tissues free of AdoMet were freeze-dried overnight and stored at −20°C until use. For AdoHcy and folates, a matrix surrogate had to be designed according to nutritive value tables [41] because of the presence of AdoHcy and folate residues in processed pig tissue. Liver surrogate consisted of 71.9% water, 20.7% lyophilized egg white, and 4.9% sunflower oil mixed with 146 mg sodium chloride, 885 mg potassium dihydrogenphosphate, and 900 mg starch. Kidney matrix was a mixture of 77.7% water, 16.9% lyophilized egg white, 3.8% sunflower oil, 379 mg sodium chloride, and 490 mg potassium dihydrogenphosphate. The brain matrix consisted of 78% water, 10.6% lyophilized egg white, 9% sunflower oil, 528 mg sodium dihydrogenphosphate, and 682 mg potassium dihydrogenphosphate. All surrogates were lyophilized overnight and stored at −20°C. Plasma surrogate for Hcy analysis was produced as published recently [13]. LC-MS/MS analysis of all blank matrices was performed to confirm the absence of all analytes. Determination of LODs and LOQs was carried out according to the procedure proposed by Hädrich and Vogelgesang [42]. For the determination of the LODs and LOQs, the analyte-free matrices were spiked with the respective folates or one-carbon metabolites at four different concentration levels starting slightly above the estimated LOD and covering one to tenfold amounts of analyte. Extraction and LC-MS/MS analysis was carried out as described above.

#### Precision of stable isotope dilution assays

Inter-assay precision was calculated by analyzing lyophilized tissue samples of female C57BL/6N mice (9 weeks) and frozen plasma in triplicate during two weeks.

Intra-assay precision was determined by analysing one sample of each tissue and plasma, respectively. CVs were calculated from triplicate injection for each sample.

#### Recoveries of stable isotope dilution assays

Blank matrices and surrogates were spiked in triplicate with three different analyte levels to verify linearity. Level I and III were calculated as the highest and lowest amounts expected in the samples. Level II was calculated from the means of all samples measured from control mice. All samples were analyzed by SIDA as described above. The recovery was calculated from the mean of the addition experiments.

In addition to the spiking experiments, tHcy in human plasma was analyzed according to the extraction procedure shown above to evaluate the validity of the plasma method. Aliquots of 50 μL of National Institute of Standards and Technology (NIST) 1955 Standard Reference Material (Level I–III) were extracted and analyzed via LC-MS/MS.

#### Data analysis

Data analysis was carried out using Xcalibur Software vers. 2.0 (Thermo Scientific, Waltham, USA) and Analyst Software vers. 1.6.2 (AB Sciex, Foster City, CA, USA). NMR spectra were processed using TopSpin 3.0 software (Bruker, Billerica, Mass., United States).

#### Statistical analysis

Linearity of response functions was performed by the Mandel-Test. Two-sided *t*-test (P = 0.05) for significance was performed with Microsoft Excel 2013.

## Results

To analyze folate along with AdoMet/AdoHcy content from the same tissue sample, both methods had to be combined. For folate extraction, enzymatic digestion of the polyglutamate forms was necessary, whereas immediate protein precipitation was essential for AdoMet and AdoHcy extraction. The presence of liquid water increased enzymatic activity and consequently resulted in alterations in metabolite composition.

### Sampling procedure

As outlined before, folate and AdoMet/AdoHcy analysis had to be carried out separately because of specific method requirements. Conventional tissue sampling and homogenization via cryo-milling or comminution in a pre-cooled mortar are time-consuming methods as each sample has to be processed one by one. Lyophilization offers a more effective and suitable method as >50 tissue samples can be transferred to a more stable condition overnight. The removal of water during the drying process leads to enhanced storage stability as it prevents enzymatic modification of most analytes depending on the presence of liquid water. Therefore, results obtained from partly thawed hydrated samples will not reflect the physiological concentrations. Moreover, homogenization of lyophilized organs is facilitated by the handling of flash-frozen samples. Particularly, small organs with 0.1 g wet weight, such as the heart and kidney, undergo simplified preservation and homogenization compared to cryo-milling as they can be homogenized in 1.5 mL Eppendorf tubes without matrix losses. Lyophilized powder was aliquoted and stored at −20°C until extraction. According to this sampling protocol, we were able to use weighed portions of 5–25 mg of tissue lyophilizate for analysis. To avoid rehydration, lyophilized samples were stored in resealable plastic bags with desiccant. Thereafter, AdoMet and AdoHcy extraction was performed at −80°C to prevent water condensation on the sample surface. The remaining tissue powder can be used for further analyses targeting genetic or metabolic information. For the validation procedure, the liver, kidney, heart, and brain were pooled after lyophilization.

### Calibration for stable isotope dilution assays

Calibration curves obtained for mixtures of isotopologic standards and analytes of 5-CH_3_-H_4_folate, 5-CHO-H_4_folate, H_4_folate, 10-CHO-folate, 5,10-CH^+^-H_4_folate, and folic acid in tissue samples were linear for the following ranges of molar ratios: 3:1–1:76 (R^2^ = 1.0000); 8:1–1:32 (R^2^ = 1.0000); 1:1–1:500 (R^2^ = 0.9997); 3:1–1:100 (R^2^ = 0.9981); 36:1–1:10 (R^2^ = 0.9965), and 4:1–1:10 (R^2^ = 0.9998), respectively. Responses for AdoMet and AdoHcy in tissue samples were linear for molar ratios from 14:1–1:24 (R^2^ = 0.9997) and 11:1–1:15 (R^2^ = 0.9998), respectively. The Hcy calibration curve for plasma was linear from 3:1–1:30 (R^2^ = 0.9993).

All response equations were calculated as follows:

A(labeled standard)/A(analyte) = RF * n(labeled standard)/n(analyte) + b

Response functions had slopes (R_F_ values) of 0.7356, 0.8861, 1.3516, 1.0667, 3.8597, 0.3803, 0.8141, 0.4517, and 0.4336 for 5-CH_3_-H_4_folate, 5-CHO-H_4_folate, H_4_folate, 10-CHO-folate, 5,10-CH^+^-H_4_folate, folic acid, AdoMet, AdoHcy, and Hcy, respectively. Intercepts (b) were +0.0114, +0.0031, +0.0005, −0.006, −0.4727, −0.0175, −0.0208, +0.005, and +0.0047, respectively.

### Optimization and validation

#### Influence of the homogenization procedure on AdoMet, AdoHcy and H_4_folate levels

**Fig 1:**
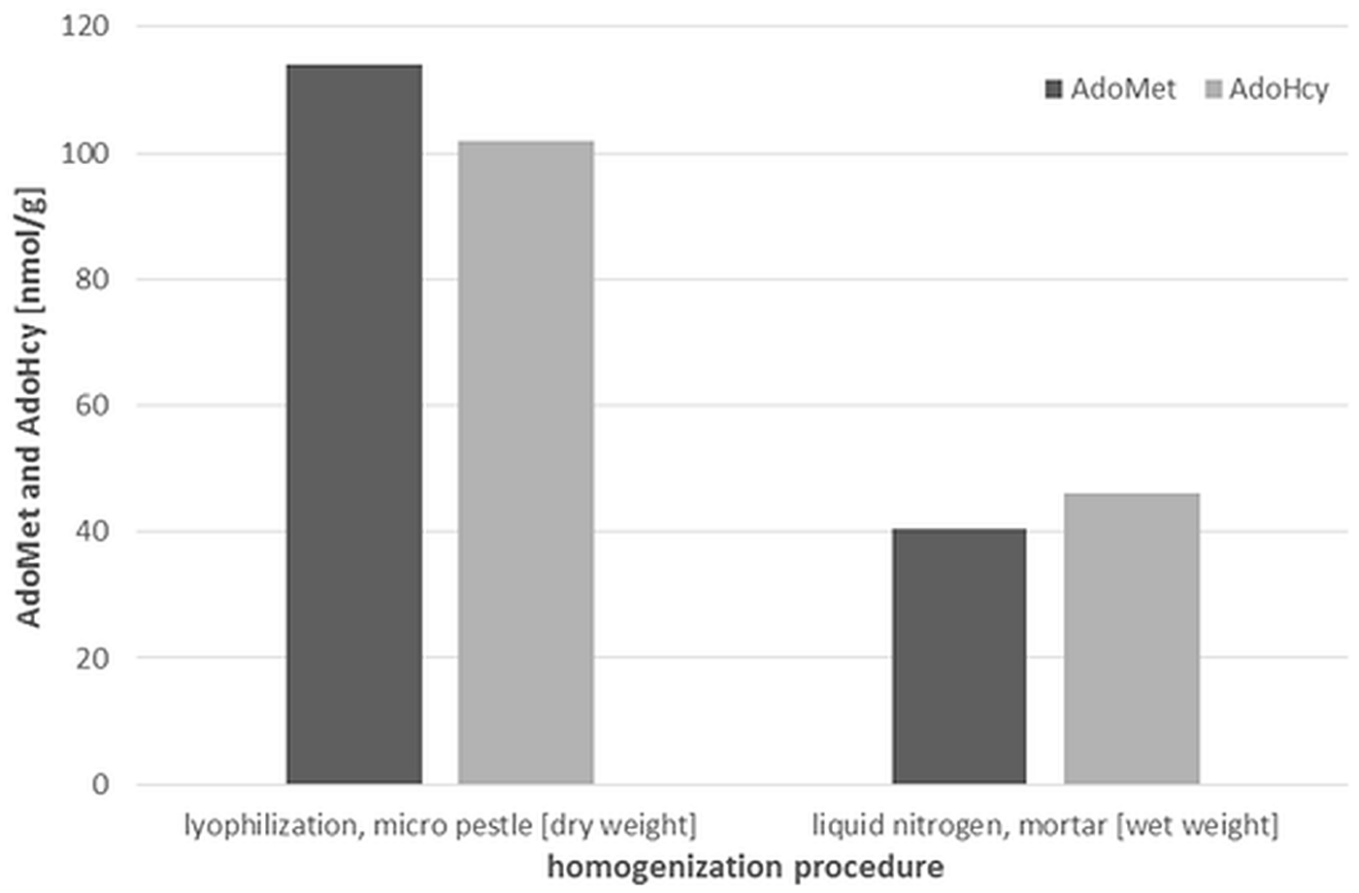
AdoMet and AdoHcy concentrations in liver after lyophilization and conventional homogenization with liquid nitrogen and cryo-milling.

We obtained 114 nmol/g dry weight (dw) and 101 nmol/g dw of AdoMet and AdoHcy, respectively, after extraction of freeze-dried samples compared to 40.4 nmol/g wet weight (ww) and 46 nmol/g ww, respectively, in conventionally prepared samples using liquid nitrogen and cryo-milling (Fig 1).

For the latter, the AdoMet/AdoHcy ratio slightly decreased from 1.1 to 0.9, which may be attributed to incomplete homogenization as debris were used and the liver was not entirely homogenized.

H_4_folate, representing the most unstable vitamer in our study, was not influenced by the overnight lyophilization step. For H_4_folate we obtained 94.4 nmol/g dw in the lyophilized liver sample and 33 nmol/g fresh weight (fw) in the conventionally homogenized sample. According to ref. [43], the dry to wet weight ratio of liver is 27.8. If converted to dry weight by multiplication by a factor of 3.6, the concentration obtained from fresh liver would be 119 nmol/g. As we used three separate parts of the liver, different analyte distributions in the tissue might contribute to the discrepancy of 20% compared to the value of the lyophilized sample. As no change in AdoMet/AdoHcy or H_4_folate was observed, lyophilization as compared to cryo-milling offered a high-throughput sampling alternative that facilitated water-free storage of organs.

#### Optimization of folate extraction

**Fig 2:**
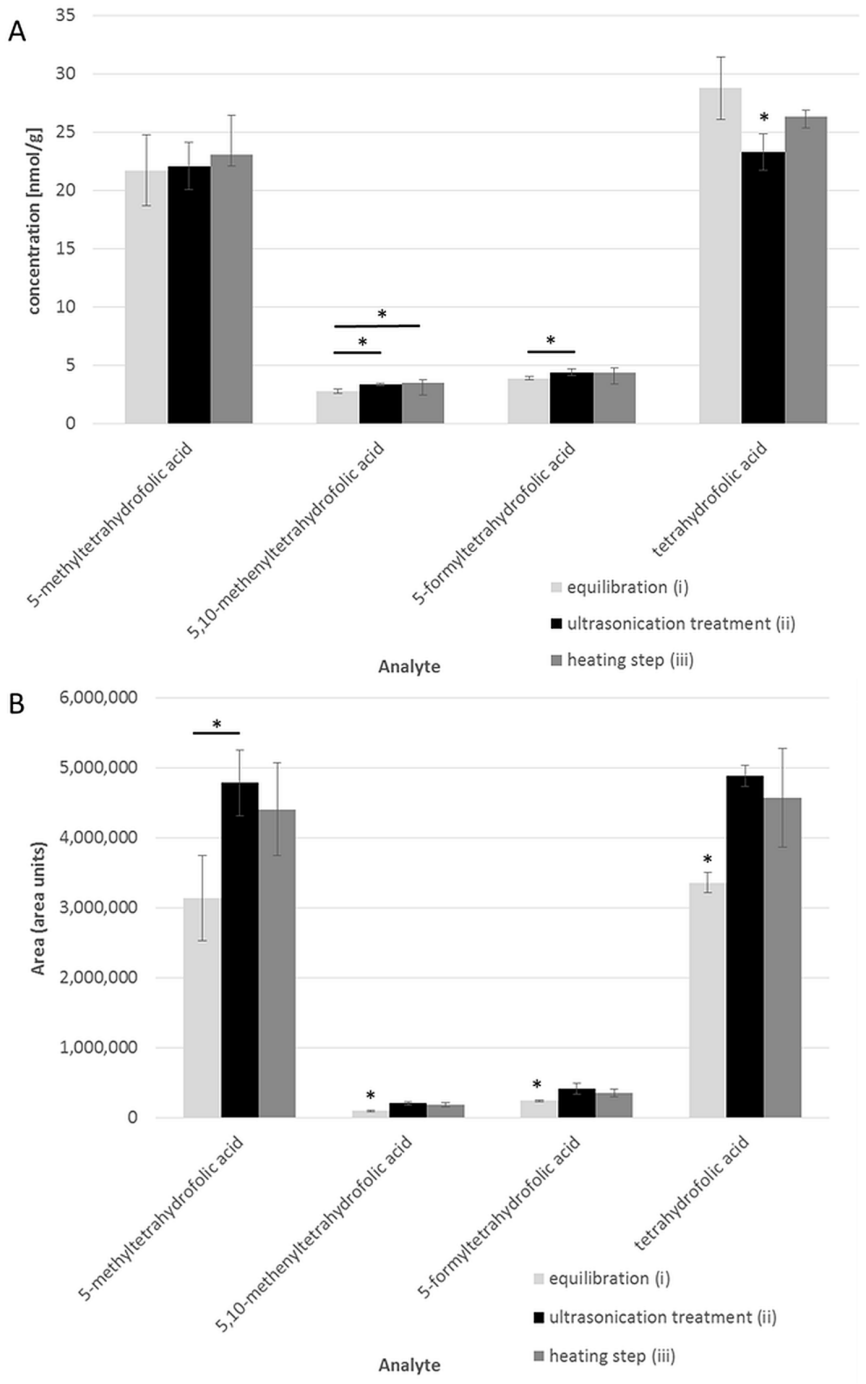
Efficiency of extracting folate vitamers in liver after different extraction and homogenization procedures. (A) Concentrations of folate vitamers in liver after different extraction procedures. Aliquots of liver were mixed with the internal standards, homogenized in 2 mL of extraction buffer 1 (without Triton X-100), and equilibrated without further treatment for 15 min (i), homogenized in extraction buffer 2 (containing Triton X-100) and subjected to ultrasonication treatment for 30 min (ii), homogenized in extraction buffer 1 and heated for 5 min in a water bath (iii). (B) Sensitivity of folate detection indicated by signal areas depending on the homogenization step. *Significant difference between extraction procedures (P < 0.05, two-sided t-test).

Three aliquots of liver were extracted in triplicate to evaluate folate interconversion during extraction. All aliquots were spiked with the deuterated internal standards and homogenized with a TissueRuptor^®^ prior to extraction. The first aliquot was equilibrated for 15 min in extraction buffer 1 (without Triton X-100). A further aliquot was suspended in extraction buffer 2 (containing Triton X-100) and placed in a chilled ultrasonic bath for cell lysis for 30 min (ii). The remaining aliquot was heated for 5 min in a water bath (iii). Afterwards, all samples (i–iii) were incubated and purified as aforementioned. The results are shown in Fig 2A.

We did not observe any interconversion between all analytes studied in this experiment or any influence of Triton X-100 on enzymatic activity during incubation. 5-CH_3_-H_4_folate contents were unaffected by treatment (i)–(iii). Significantly lower levels of 5,10-CH^+^-H_4_folate were observed in untreated samples than in samples undergoing ultrasonication or cooking (*P* < 0.05, two-sided *t*-test). Furthermore, less 5-CHO-H_4_folate was found in these samples than in samples after ultrasonication. The results for H_4_folate ranged from 23 nmol/g dw for ultrasonic treatment to 28 nmol/g dw for 15 min of equilibration, with a significantly decreased mean for the ultrasonicated samples. Despite significant differences, small errors from the weighing process have to be pronounced. Because the weighed portions were <8 mg, small variations might contribute to these findings.

Nevertheless, we obtained significantly higher areas and sensitivity for all analytes after ultasonication treatment or cooking (except for 5-CH_3_-H_4_folate) than non-treated samples (Fig 2B).

For the heating step, we obtained higher standard deviations than for the ultrasonication treatment. Therefore, we chose the ultrasonication treatment as the most suitable extraction method for our assay.

#### Influence of DTT and formic acid on AdoMet during extraction and detection

AdoMet is generally accepted as an analytically challenging compound because of its enzymatic interconversion to AdoHcy in raw material or its degradation in purified samples. Acids like acetic acid are commonly used to stabilize AdoMet in plasma samples [28,44,45]. Gellekink and coworkers [28] investigated the influence of acetic acid on plasma AdoMet stability and found a decrease of AdoMet and a parallel increase of AdoHcy in non-acidified samples even at −20°C. As we used a sample concentration procedure with nitrogen, a more volatile acid was needed to avoid excess acidification, which could also impair AdoMet stability. Therefore, we analyzed the stability of AdoMet in the presence of formic acid. Furthermore, we evaluated DTT as a reductive additive and its preservative effect on both analyte and sensitivity during LC-MS/MS measurement. To investigate the suitability of both additives, six aliquots of 10 mg liver surrogate were spiked with known amounts of AdoMet. For solvent (i) with DTT and formic acid and (ii) without DTT, we obtained recoveries of 61% and 55%, respectively, in comparison with only 46% for solvent (iii) without additives.

**Fig 3:**
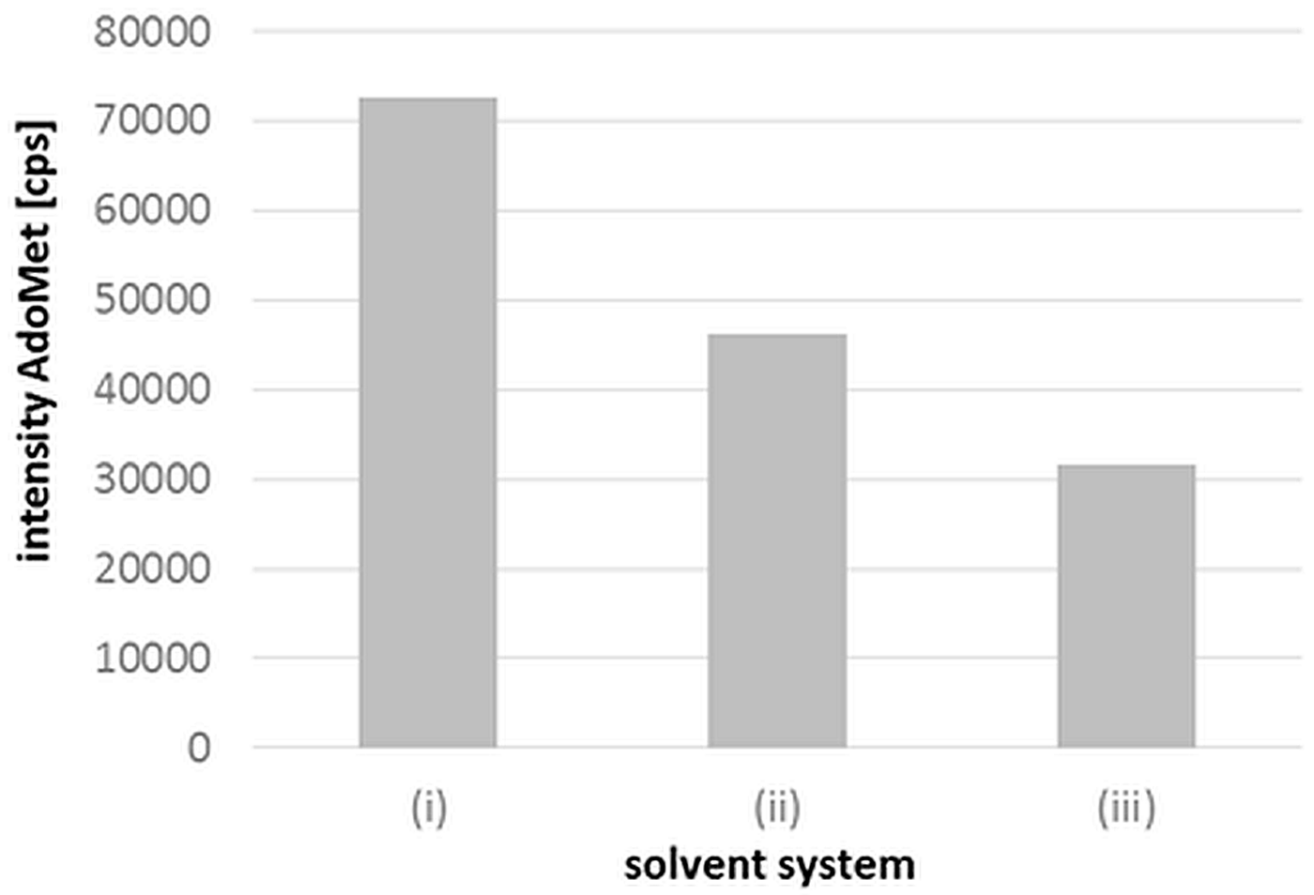
Influence of different solvent mixtures on AdoMet extraction and detection. Cps: counts per second. (i) methanol/water (90/10 v/v) + DTT + 0.1% (v/v) formic acid, (ii), methanol/water (90/10 v/v) + 0.1% (v/v) formic acid, (iii) methanol/water (90/10 v/v).

As AdoMet has a retention time of 4.5 min, matrix interferences and signal suppression likely decreased the intensity of AdoMet, resulting in higher LODs and LOQs. Therefore, minimized analyte losses are necessary for sensitive AdoMet detection. After extraction with solvent (i), we observed higher signal intensities for AdoMet (Fig 3) than for solvent system (ii) and more than twofold higher signals than for system (iii).

This underlines the necessity of an acidic and reductive environment for AdoMet. Therefore, we chose solvent mixture (i) for sample extraction.

#### Optimal DTT concentration for Hcy reduction

The addition of a more concentrated aqueous DTT solution (400 mmol/L) did not improve Hcy extraction by further stabilization against oxidation during the drying process under nitrogen flow compared to a 200 mmol/L solution (data not shown). Therefore, adding 50 μL of a 200 mmol/L solution to 50 μL of plasma, giving a final concentration of 100 mmol/L DTT was sufficient to reduce bound and oxidized Hcy, as previously described by Nelson and coworkers [37].

#### Analysis of AdoMet and AdoHcy in tissue samples

For the determination of LOD/LOQ for AdoMet, a matrix free from the analyte had to be prepared. Therefore, pig liver, kidney, and brain were treated with hydrogen peroxide as mentioned above and lyophilized overnight. After the lyophilization step we obtained an anhydrous matrix free of measurable AdoMet residues with similar physical properties compared to the lyophilized mouse tissue. Because AdoHcy residues were still detectable after this treatment these matrices were not suitable for LOD/LOQ or recovery determination of the latter. The surrogates of pig liver, kidney, and brain for AdoHcy were a mixture of the five major constituents, water, protein, fat, and minerals according to [41]. The LOD/LOQ and recovery of each analyte in the heart was calculated from the surrogate with similar validation values, i.e., kidney.

Using the procedure by Hädrich and Vogelgesang [42], we determined the LODs and LOQs shown in Table 3.

**Table 3:** LODs and LOQs for folates and other one-carbon metabolites in tissue and plasma.

Compound	LOQs and LODs in tissue [nmol/g dw] and plasma [nmol/L]									
	Liver		Kidney		Heart		Brain		Plasma	
	LOD	LOQ	LOD	LOQ	LOD	LOQ	LOD	LOQ	LOD	LOQ
AdoMet	4.66	13.9	3.05	9.11	3.05	9.11	1.68	5.01		
AdoHcy	0.39	1.14	0.45	1.32	0.32	0.95	0.16	0.48		
tHcy									50.0	149
H_4_folate	0.26	0.77	0.17	0.49	0.03	0.10	0.05	0.16		
5-CH_3_-H_4_folate	0.19	0.55	0.07	0.21	0.01	0.04	0.01	0.04		
5-CHO-H_4_folate	0.06	0.19	0.24	0.71	0.05	0.14	0.02	0.06		
5,10-CH^+^-H_4_folate	0.19	0.56	0.08	0.25	0.05	0.13	0.05	0.13		
10-CHO-folate	0.03	0.10	0.04	0.12	0.01	0.02	0.01	0.02		
Folic acid	0.28	0.83	0.20	0.59	0.05	0.15	0.06	0.16		

The about tenfold higher LODs for AdoMet than for AdoHcy were ascribed to a higher matrix suppression of the AdoMet signal as compounds of low molecular weight elute from the HPLC-column in the expected timeframe for AdoMet contributing to a decrease in sensitivity. Injection analysis of the matrix via HPLC and the analyte via the syringe pump in analogy to the procedure reported recently [40] confirmed these findings (data not shown). AdoHcy signal intensity was not impaired by matrix interferences, and, thus, a sensitive detection was ensured.

The recoveries of AdoMet and AdoHcy in tissue were determined by spiking the pig tissue blank or surrogate with three different amounts of the respective analyte and performing SIDA with SPE clean-up as detailed before. All recoveries obtained from the spiking experiments are shown in Table 4.

**Table 4:** Recoveries of the SIDA for folates and other one-carbon metabolites in tissue and plasma.

Compound	Recoveries in tissue and plasma [%]														
	Liver			Kidney			Heart			Brain			Plasma		
Spiking level	I	II	III	I	II	III	I	II	III	I	II	III	I	II	III
AdoMet	109	98	102	95	101	109	95	101	109	100	97	102			
AdoHcy	101	101	98	95	110	95	94	93	95	94	93	95			
tHcy													106	100	102
H_4_folate	107	97	104	102	101	105	107	104	97	107	104	97			
5-CH_3_-H_4_folate	109	101	95	112	107	98	97	93	93	97	93	93			
5-CHO-H_4_folate	109	108	116	102	111	101	110	110	111	110	110	111			
5,10-CH^+^-H_4_folate	100	89	98	77	98	n/s	83	116	n/s	83	116	n/s			
10-CHO-folate	106	108	94	99	99	95	95	92	108	95	92	108			
Folic acid	106	104	n/s	89	101	99	98	97	n/s	98	97	n/s			

n/s: not specified.

**Fig 4:**
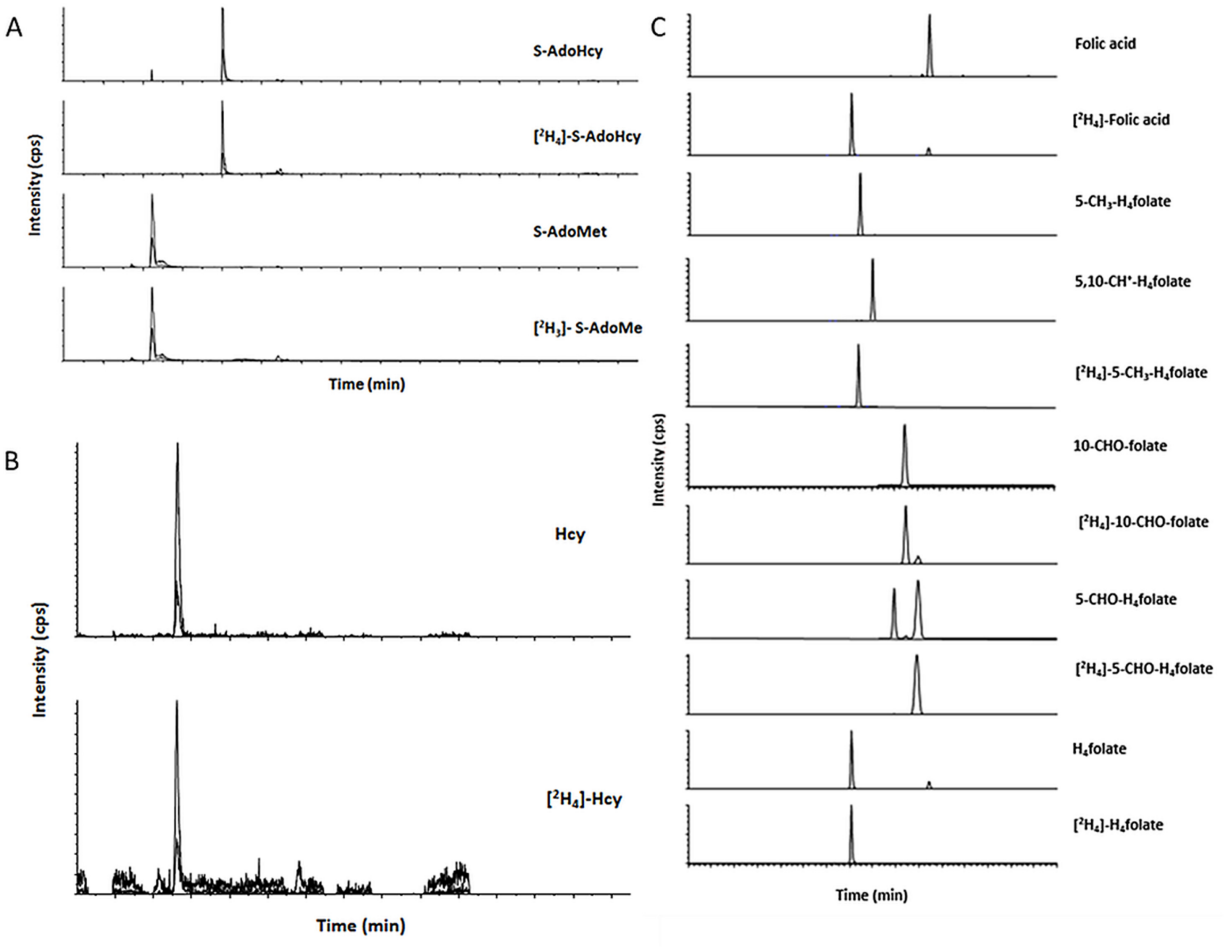
Characteristic MRM chromatograms for (A) AdoMet and AdoHcy in tissue, (B) for tHcy in plasma, (C) for folate vitamers in tissue.

All recoveries ranged from 95–109% and from 93–110% for AdoMet and AdoHcy, respectively, confirming the accuracy of the method. A typical MRM chromatogram for tissue analysis is shown in Fig 4A.

#### Analysis of tHcy in plasma

Only ~1% of Hcy occurs in its free form in plasma. The major part is bound to proteins, cysteine, or another Hcy molecule forming homocystine [38]. Therefore, a successful tHcy determination requires an appropriate amount of reduction agent. As most methods for tHcy analysis use DTT, we included this reductant in our method. Different reduction conditions with varying DTT concentrations in plasma have been published by Nelson and coworkers [37]. They found a final concentration of 100 mmol DTT/L plasma to be the most suitable for complete and instantaneous disulphide bond reduction [37]. We adapted this DTT/plasma ratio for our method, as well as a 15 min reduction time for Hcy. Whereas most of the previously reported methods have been optimized for human plasma, we had to decrease the sample amount to smaller volumes as mice give only a small amount of blood specimen. To enable further screenings, we decided to process 50 μL of mouse plasma in our method. Amounts <50 μL lacked reproducibility as the signals for analyte and standard were not very intense.

According to the procedure of Hädrich & Vogelgesang [42], we determined 50.0 and 149 nmol/L as the LOD and LOQ for tHcy (Table 2). These concentrations are 60- and 20-fold lower than the expected sample concentrations for C57BL/6N mice, thereby confirming that 50 μL of plasma was sufficient for sensitive tHcy screening. Compared to Li and coworkers [26], who determined 0.5 and 5 μmol/L as the LOD and LOQ, our method had an increased sensitivity by a factor of 10 and 33 for LOD and LOQ, respectively. This might be partly attributed to the additional centrifugation step of our method after the evaporation step leading to a higher purity of the extracts. A dilution of deproteinated pooled human plasma is often used as zero blank for validation. In comparison, our surrogate mimics the plasma matrix in a more realistic way as comparable amounts of matrix are present during extraction.

The recoveries for spiked plasma surrogates were 106%, 100%, and 102% for spiking level I, II, and III, respectively (Table 3). For further verification, tHcy in human plasma was analyzed according to the extraction procedure shown above. Aliquots of 50 μL of NIST Standard Reference Material 1955 (Level I–III) were extracted and analyzed in duplicate via LC-MS/MS. For level I–III with certified mean values of 3.98±0.18, 8.85±0.60, and 17.7±1.1 μmol/L tHcy, we found 3.46, 8.24, and 15.9 μmol/L tHcy comprising recoveries of 87%, 93%, and 90%, respectively. Considering the certification range (±SD), the maximum recoveries were 91%, 100%, and 96%, respectively, confirming the accuracy of our method in the concentration range of the samples. Moreover, the results of our method for NIST 1955 SRM are comparable to the results of the liquid chromatography coupled with fluorometric detection (LC-FD) method of the Centers for Disease Control and Prevention (CDC) [46] with 3.5±0.3, 8.2±0.6, and 17±1 μmol/L tHcy for level I–III giving corresponding recoveries of 99%, 100%, and 94%, respectively. As we did not purify the plasma samples by anion exchange solid phase extraction, our method offers a fast and solid alternative compared to the methods used by NIST. A typical chromatogram is shown in Fig 4B.

The equivalence of methods and their diagnostic validity has also been evaluated by Nelson and coworkers [47], who compared LC-MS, LC-MS/MS, gas chromatography coupled with mass spectrometry (GC-MS), fluorescence polarization immunoassay (FPIA), and LC-FD. All of these methods have been found to have similar results for tHcy and, therefore, applicable for clinical assessment [47].

#### Analysis of folate in tissue samples

Folates are ubiquitous in tissue and blood; the liver and kidney contain major amounts of folate. Therefore, the tissue surrogates used for the validation of AdoHcy were also applied to determine LODs, LOQs, and recoveries for folates. Table 2 shows the tissue specific LODs and LOQs for all folates studied. All values were sufficiently low to determine all bioactive folate forms except 5,10-CH^+^-H_4_folate in brain. The recoveries for all analytes ranged from 97–107% for H_4_folate, 93–112% for 5-CH_3-_H_4_folate, 101–116% for 5-CHO-H_4_folate, 92–108% for 10-CHO-folate, and 89–106% for folic acid. The highest variations were found for 5,10-CH^+^-H_4_folate with recoveries from 77–116%. The determination of the recoveries for the latter was challenging because of steady interconversion of the vitamer to 5-CHO-H_4_folate [48]. As 5-CHO-H_4_folate is the only hydrolysis product in the presence of reducing agents [48], slightly higher levels of this formylated vitamer were determined when assessed together with 5,10-CH^+^-H_4_folate. For the precise determination, sample measurements have to be carried out with repeated measurements of analyte/standard mixtures to compensate for analyte degradation. For folic acid in the liver, heart, and brain and 5,10-CH^+^-H_4_folate in the kidney, heart, and brain, spiking level III was not determined as all sample concentrations were expected to be slightly above the LOQs or even lower (Table 2). The newly developed method was based on the groundwork of Mönch and coworkers [13] for the determination of folate vitamers in erythrocytes. We have already succeeded in minimizing the described extraction procedure for folate determination in dried blood spots [49]. As the amount of matrix is comparable to that for the dried blood spots, we decided to adopt the extraction procedure with minor modifications. Advantages of the additional lyophilization step have already been mentioned above. A typical MRM chromatogram for tissue folate is shown in Fig 4C.

#### Precision of the new assays

Intra-assay precision was determined by multiple injections (n = 3) of one tissue extract prepared within one day. Inter-assay precision was determined by extraction of samples within two weeks. All analyses were performed in triplicate. Results for the multi-injection assay and the inter-assay studies are shown in Table 5.

**Table 5:** Intra- and inter-assay precision for tissue and plasma analysis.

Compound	Intra- and inter-assay precision [%]							
	Liver		Kidney		Brain		Plasma	
	intra	inter	intra	inter	intra	inter	intra	inter
AdoMet	0.9	8.7	1.8	5.9	1.4	5.6		
AdoHcy	4.9	8.2	1.7	7.4	2.0	7.6		
tHcy							2.2	11.6
H_4_folate	6.3	11.4	4.6	14.7	10.4	27.5		
5-CH_3_-H_4_folate	1.8	13.5	1.1	11.2	6.7	10.3		
5-CHO-H_4_folate	2.0	1.9	0.9	15.0	1.8	23.2		
5,10-CH^+^-H_4_folate	5.2	7.0	11.0	12.2	<LOQ	<LOQ		
10-CHO-folate	14.4	18.8	11.6	29.5	3.6	27.1		
Folic acid	<LOD	<LOD	21.1	13.5	<LOQ	<LOQ		

Intra: intra-assay precision; inter: inter-assay precision.

For AdoMet, AdoHcy, and tHcy, low intra- and inter-assay variations were achieved despite low sample input. The slightly increased inter-assay variation for tHcy was attributed to small variations in the pipetted volume of 10 μL of plasma. Results for folates showed precise intra-assay coefficients of variation (CVs) for all vitamers except for 10-CHO-folate in the liver or folic acid in the kidney. The latter was attributed to the small weighed portions of 5 mg liver homogenate and 10 mg kidney homogenate resulting in low intensities of the signals. Therefore, higher variations can be expected for the signal areas leading to higher CVs for intra- and inter-assay precision. The concentration of folic acid in the kidney was only threefold higher than the LOQ, thus leading to higher variations in the multi-injection assay.

#### Applications of the multi stable isotope dilution assay

To demonstrate the applicability of the developed methods, three standard inbred C57BL/6N mice (9 weeks) were examined for their folate patterns and AdoMet/AdoHcy status in tissue and tHcy in plasma. Table 6 shows the results for all analytes studied.

**Table 6:** Folate, AdoMet, and AdoHcy in tissue and tHcy in plasma of C57BL/6N mice (female, 9 weeks).

Compound	Analyte concentration in tissue [nmol/g dw] and plasma [nmol/L]				
	Liver	Kidney	Heart	Brain	Plasma
AdoMet	129	90.8	108	85.3	
AdoHcy	91	61.8	10.6	21.6	
tHcy					2893
H_4_folate	123	23.7	1.43	1.05	
5-CH_3_-H_4_folate	43.9	53.7	2.1	2.75	
5-CHO-H_4_folate	24.2	6.54	0.42	0.41	
5,10-CH^+^-H_4_folate	12.8	2.91	0.32	<LOQ	
10-CHO-folate	0.51	6.82	0.08	0.28	
Folic acid	<LOD	1.41	<LOD	<LOQ	

The highest amounts of AdoMet were found in the liver and heart with 129 and 108 nmol/g dw, respectively, followed by the kidney and brain with 90.8 and 85.3 nmol/g dw, respectively. Whereas the AdoMet values ranged between 85.3 and 129 nmol/g dw, we found a higher discrepancy between the tissue AdoHcy values, which ranged from 10.6 and 21.6 nmol/g dw for the heart and brain to 61.8 and 91 nmol/g dw for the kidney and liver. In the plasma, we found 2.89 μmol/L tHcy. The results obtained for the liver were similar to the results for C57BL/6N control mice from Dahlhoff et al. [33], also using a SIDA. For the conversion of the metabolite tissue concentrations between ww and dw, a tissue specific factor of 3.6 (for liver, shown above) has to be included. For assessing tissue folate, we determined the concentrations of the bioactive forms 5-CHO-H_4_folate, H_4_folate, 5-CH_3_-H_4_folate, and 5,10-CH^+^-H_4_folate, and the inactive, oxidized forms 10-CHO-folate and folic acid. The highest amounts of folate were found in the liver and kidney in which H_4_folate with 123 and 23.7 nmol/g dw and 5-CH_3_-H_4_folate with 43.9 and 53.7 nmol/g dw represented the main folate vitamers. Tissue levels in the brain and heart were twentyfold lower than in the kidney with 1.05 and 1.43 nmol H_4_folate/g dw and 2.75 and 2.1 nmol 5-CH_3_-H_4_folate/g dw, respectively. Significant amounts of 5-CHO-H_4_folate, with 24.2 nmol/g dw and 6.54 nmol/g dw, and 5,10-CH^+^-H_4_folate, with 12.8 nmol/g dw and 2.91 nmol/g dw, were found in the liver and kidney.

## Discussion

Combined analysis of selected one-carbon metabolites from one tissue sample allows a closer insight into the tissue distribution of folate vitamers and the methylation capacity mirrored by AdoMet and AdoHcy status. Disagreement among recent publications on one-carbon metabolite distribution in tissue samples of control mice, e.g., in the liver, ranging from approximately 40 [33] to 126 nmol/g [32] for AdoMet and from approximately 28.4 [32] to 40.7 nmol/g [36] for AdoHcy, can be attributed either to different mouse types studied with differing enzyme expression of one-carbon metabolism or to divergence in feeding or methodologies applied for analyses. In particular, different tissue sampling techniques and storing conditions lead to alterations in AdoMet and AdoHcy content in tissue and furthermore to a certain discrepancy of AdoMet/AdoHcy ratios as recently shown [31,36]. Most methods use applications coupled with UV detection for the quantitation of AdoMet and AdoHcy in mouse tissue [32,50–53] and amperometric detection [54], electrochemical detection [55], or microbiological assays [56–58] for folate analysis.

Because of their sensitivity (subnanogram levels) and similar response to most folate vitamers microbiological assays measuring the turbidity of microbial growth on folate extracts have been regarded as ´gold standard´ for decades [59]. Nevertheless, microbiological assays cannot distinguish between folate vitamers and growth factors or inhibitors originating from the matrix significantly influence bacterial growth [59]. Within the last two decades, SIDAs have proven their advantages with respect to accuracy and precise quantitation [60] and allow complex studies of folate vitamers in combination with LC-MS/MS. AdoMet and AdoHcy underlie enzymatic or chemical conversion leading to an artefactual AdoMet/AdoHcy ratio. Internal standards compensate for the interconversion and the losses of analyte.

AdoMet plays an important role in the transfer of one-carbon moieties in the methylation of DNA, proteins, phospholipids, and other molecules like neurotransmitters [22]. After the methylation reaction, AdoHcy is metabolized to Hcy and is then either converted to cystathionine in the trans-sulfuration pathway or serves as a methyl acceptor for the remethylation step to methionine [22]. As both AdoMet and AdoHcy are ubiquitous in tissue and surrogate, an analyte-free matrix was not available and had to be developed for recoveries and LOD/LOQ. Compared to the assay of Krijt and coworkers [36], we obtained a comparable LOD for AdoMet but a 38-fold lower LOD for AdoHcy, thereby underlining the impact of matrix constituents on the AdoMet signal.

Regarding recoveries (ranging between 93% and 110%) and precision, the method presented here also revealed excellent validation data. Compared to other methodologies with recoveries of 98.4%–104.9% [36], the higher variations of our method were because of the lower amounts of AdoMet and AdoHcy spiked to the matrix surrogate. In comparison to methods using cartridge extraction [29], we were able to minimize material costs because of the smaller amount of chemicals used. Compared to the methodology of Krijt and coworkers [36], who used 50–150 mg wet weight (ww) of liver or kidney, we were able to achieve a three- to tenfold lower sample input after converting our values from dw to ww. According to the estimated dry to wet weight ratio of 27.8% reported in [43], the conversion factor would be 3.6 for liver. For protein precipitation and acidification, perchloric acid is commonly used [36,61], and perchloric acid residues have to be precipitated with potassium carbonate after incubation. In contrast to this, lyophilizates do not require immediate perchloric acid treatment as no water is present in the matrix. The cooling mixture with dry ice at and acetone at −80°C combined with methanol/water (90/10, v/v) ensured fast precipitation of enzymes and further proteins in the powder, thus preventing enzymatic AdoMet conversion. As detailed before, DTT and formic acid lead to further stabilization of the analyte. A similar constitution of the precipitation agent is used by Korinek and coworkers [62] for the determination of AdoMet and AdoHcy in cells [62]. After precipitation and centrifugation, we used nitrogen gas to evaporate methanol in analogy to ref. [63] for sample concentration followed by resolving the pellet in the initial mixture of the mobile phase to avoid interferences and unfavorable peak shapes or shifts.

Currently, microsampling techniques with low sample volumes and weights are in great demand. For tHcy analysis in plasma, we decreased the sample volume to 50 μL of plasma. tHcy determination in plasma revealed diagnostic validity when compared to the NIST and CDC methods. By introducing a lyophilization step before homogenization and extraction, we were able to decrease sample input. For tissue, we used 5 to 25 mg lyophilizate for extraction to increase the cost-effectiveness and minimize the time required for the method.

## Conclusion

Multi SIDAs combined with LC-MS/MS analysis are advantageous when compared to microbiological assays or methods using UV spectroscopic, amperometric, or electrochemical detection. To bridge the gap between various methods published for AdoMet/AdoHcy, tHcy, or folate analysis, we developed three highly sensitive and solid methods for the determination of these analytes in one tissue sample and plasma while simultaneously meeting the requirements of both analyte groups. Small sample amounts (5–25 mg) of tissue and 50 μL of plasma decreased the amount of chemicals used and the time needed for extraction. The lyophilization step offered the opportunity to process large sample amounts overnight and enabled repeated freeze-thaw cycles. The remaining lyophilizates can be applied to additional metabolite screenings. Recommended applications for these methods are deficiency and fortification studies in animals for folate and vitamin B_12_, which could create a general overview on tissue-specific folate patterns and the impact on AdoMet/AdoHcy and tHcy levels.
